# gymnotoa-db: a database and application to optimize functional annotation in gymnosperms

**DOI:** 10.1093/database/baaf019

**Published:** 2025-03-06

**Authors:** Fernando Mora-Márquez, Mikel Hurtado, Unai López de Heredia

**Affiliations:** GI en Desarrollo de Especies y Comunidades Leñosas (WooSP), Dpto. Sistemas y Recursos Naturales, ETSI Montes, Forestal y del Medio Natural, Universidad Politécnica de Madrid, José Antonio Novais 10, Madrid 28040, Spain; GI en Desarrollo de Especies y Comunidades Leñosas (WooSP), Dpto. Sistemas y Recursos Naturales, ETSI Montes, Forestal y del Medio Natural, Universidad Politécnica de Madrid, José Antonio Novais 10, Madrid 28040, Spain; GI en Desarrollo de Especies y Comunidades Leñosas (WooSP), Dpto. Sistemas y Recursos Naturales, ETSI Montes, Forestal y del Medio Natural, Universidad Politécnica de Madrid, José Antonio Novais 10, Madrid 28040, Spain

## Abstract

Gymnosperms are a clade of non-flowering plants that include about 1000 living species. Due to their complex genomes and lack of genomic resources, functional annotation in genomics and transcriptomics on gymnosperms suffers from limitations. Here we present gymnotoa-db, which is a novel, publicly accessible relational database designed to facilitate functional annotation in gymnosperms. This database stores non-redundant records of gymnosperm proteins, encompassing taxonomic and functional information. The complementary software, gymnotoa-app, enables users to download gymnotoa-db and execute a comprehensive functional annotation pipeline for high-throughput sequencing-derived DNA or cDNA sequences. gymnotoa-app’s user-friendly interface and efficient algorithms streamline the functional annotation process, making it an invaluable tool for researchers studying gymnosperms. We compared gymnotoa-app’s performance against other annotation tools utilizing disparate reference databases. Our results demonstrate gymnotoa-app’s superior ability to accurately annotate gymnosperm transcripts, recovering a greater number of transcripts and unique, non-redundant Gene Ontology terms. gymnotoa-db’s distinctive features include comprehensive coverage with a non-redundant dataset of gymnosperm protein sequences, robust functional information that integrates data from multiple ontology systems, including GO, KEGG, EC, and MetaCYC, while keeping the taxonomic context, including *Arabidopsis* homologs.

**Database URL**: https://blogs.upm.es/gymnotoa-db/gymnotoa-db/

## Introduction

Gymnosperms are a non-flowering seed plant clade of about 1000 living species that includes three classes (Cycadopsida, Ginkgoopsida, and Pinopsida), five subclasses (Cycadidae, Ginkgoidae, Cupressaceae, Pinidae, and Gnetidae), eight orders (Cycadales, Ginkgoales, Araucariales, Cupressales, Pinales, Ephedrales, Gnetales, and Welwitschiales), 13 families, and 86 genera [1], and that diverged from angiosperms *c*. 300–350 million years before present [2]. Most gymnosperms are long-lived woody plant species of great economic and ecological importance widely distributed around the globe. However, their life‐history characteristics, large genome sizes, and lack of genomic resources if compared to other plant model species, limit the initiatives to address biological questions and the functional evolution of gymnosperms [3]. Obtaining large sets of genomic and transcriptomic sequences from model and non-model organisms, such as gymnosperms, is a routine task nowadays due to the increase of sequencing capacity provided by high-throughput platforms [4]. Nevertheless, extracting unbiased consequential conclusions is still bottlenecked by the lack of proper bioinformatic processing of genomic information [5].

In gymnosperms, and particularly in conifers, the genome size is much larger than in other plants due to the extensive contribution of interspersed repetitive content among the *c*. 20 000–50 000 protein-coding genes identified for the different species [6]. The repetitive content in the gymnosperms genomes is the product of long-terminal retrotransposons nested within the long introns of some gene families and gene duplication [7]. For instance, while oaks have genomes with <1 Gb of sequence, gymnosperms have much larger genomes: *Ginkgo biloba* (10.6 Gb) [8], *Cycas panzhihuaensis* (10.5 Gb) [9], *Picea abies* (12.3 Gb) [10], or *Pinus taeda* (20.6 Gb) [11]. Despite this huge genome complexity, several efficient bioinformatic pipelines and algorithms facilitate processing the output of next-generation sequencing platforms [12, 13]. Moreover, the problems related to the inherent large hardware infrastructure required to accomplish this task have been partially solved by using high-performance computation clusters [14] or cloud computing [15].

Possibly, one of the main limitations is the annotation of novel genes or transcripts [16]. Functional annotation of sets of sequences obtained from next-generation sequencing experiments is usually performed by searching for homologous sequences deposited in general purpose databases such as RefSeq [17], Uniprot/Swissprot [18], or in species-specific genomic repositories (e.g. TAIR for *Arabidopsis thaliana*) [19] with BLAST+ [20] or DIAMOND [21], to further collect the Gene Ontology [22, 23], KEGG ontology [24] or metabolic pathway information related to those sequences, such as MetaCYC [25] or Reactome Pathway Knowledgebase [26]. Several general-purpose software applications have been implemented to access and process the information stored in the genomic and proteomic databases, such as Blast2GO [27], Trinotate [28], or EnTAP [29]. Relations between protein sequences and their function can be also obtained from the comprehensive databases InterPro [30] or eggNOG [31], through the specific software applications InterProScan [32] and eggNOG-mapper [33]. While InterPro provides functional analysis of protein sequences by classifying them into families and predicting domains and important sites, eggNOG is a database that holds orthologous groups of proteins and functional annotations of 12 535 organisms, including plant species.

For gymnosperms, there are some comprehensive resources, such as the recent release of a curated benchmark gene set specific for gymnosperms [34] to facilitate assessing genome and annotation completeness in BUSCO [35], or the genome-wide data, transcriptomes (cDNA, EST and TSA) and unigenes, with varying curation level for a small number of the 2026 species of forest trees included in TreeGenes [36]. Notwithstanding, obtaining high-quality genomes and annotations remains a major challenge because specific gene functional annotation information is scattered, incomplete, or with low curation level. Actually, it is very frequent to utilize genomic resources from other land plants. This approach may not be adequate, due to the far evolutionary distance between the major lineages, especially between angiosperms and gymnosperms [34]. In addition, duplication, redundancy, and inconsistency may undermine the accuracy of analyses undertaken on bioinformatics databases [37].

Plant-oriented taxonomy aware annotation tools, such as TRAPID [38] and TOA [39], make use of the PLAZA data set [40] and produce improved annotation results over other general purpose application tools. PLAZA provides a curated and comprehensive comparative genomic resource for green plants, including annotated proteins, GO/KEGG terms, and InterPro domains, among others. PLAZA has instances specifically focused on gymnosperms, monocot and dicot plants, respectively [41]. The PLAZA instance for gymnosperms, PLAZA Gymno01, integrated structural and functional annotation of 16 species and included 777 165 genes of which 97.2% were protein coding. These protein-coding genes were clustered in 30 041 multi-gene gene families (48.3% multi-species gene families), resulting in 14 819 phylogenetic trees. Unfortunately, PLAZA Gymno01 database is no longer maintained.

In the present manuscript, we introduce gymnotoa-db, an up-to-date comprehensive relational database that includes curated records of gymnosperm proteins and related taxonomic and functional information. The companion software gymnotoa-app is distributed together with gymnotoa-db, so as to allow easy database download and running a full functional annotation pipeline for high-throughput sequencing-derived DNA or cDNA sequences. gymnotoa follows the spirit of PLAZA Gymno01 and TOA taxonomy-aware functional annotation tool and is intended to be a public reference database to help producing accurate functional annotation reports in genomics and transcriptomics experiments on gymnosperm species. We provide evidence of the robustness of gymnotoa-db records by analyzing the completeness and lack of redundancy of protein sequences and associated functional information in the database. Furthermore, we demonstrate the efficiency of gymnotoa-app by comparing its performance against other annotation tools that employ different databases using a benchmark transcriptome file from a pine species and a gold-standard transcriptome built from experimentally tested proteins and robust-associated functional information.

## Material and methods

### gymnoTOA-DB database implementation and maintenance

**Figure 1.**
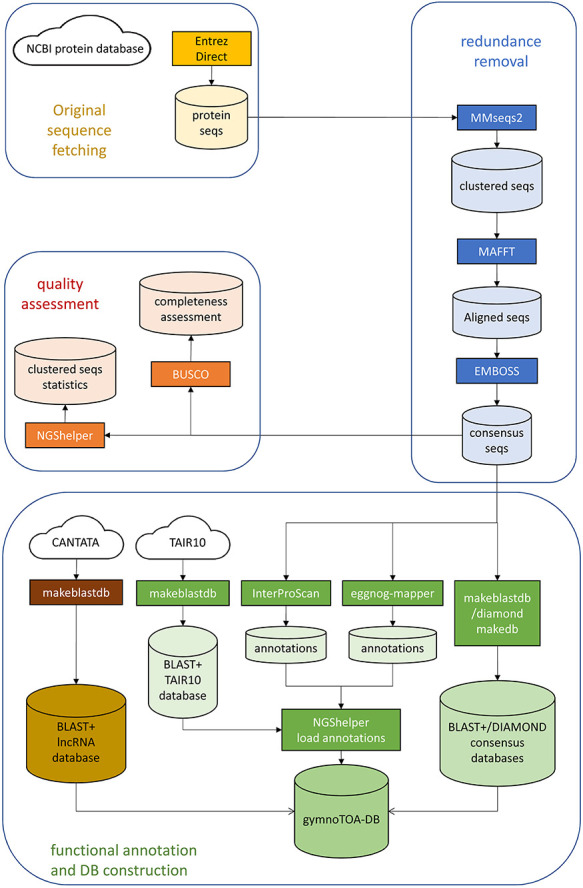
GymnoTOA-DB construction workflow.

The workflow to fetch, process, and build gymnotoa-db consists of instructions encapsulated in a single BASH script, build-gymnotoa-db.sh, available with gymnotoa-app distribution at GitHub (https://github.com/GGFHF/gymnoTOA-app/tree/main/database-building), that includes the steps as follows (Fig. 1). Protein sequences are automatically collected from the NCBI Protein database for Acrogymnospermae using EntrezDirect [42]. The Protein database from NCBI is a collection of sequences from several sources, including translations from annotated coding regions in GenBank, RefSeq, and TPA, as well as records from UniProt/SwissProt (https://www.uniprot.org/), PIR (https://www.prf.or.jp/index-e.html), PRF (https://proteininformationresource.org/), and PDB (https://www.rcsb.org/). Clustering of all the fetched protein records and sequences to avoid within and between taxa redundancy is performed using MMSEQS2 [43]. Clusters keep the taxonomic information of the original protein sequences.

The optimal MMSEQS2 parameters were assessed so as to get the smallest consensus protein datasets without losing functional information. To quantify and remove those sequences with redundant functional information, we used MMSEQS2, testing several combinations of sensitivity (s), minimum coverage (c) and minimum sequence identity (—min-seq.id) (Table 1). For each parameter combination, the average percentage of identity between the consensus sequence and the original sequences forming the cluster was calculated. In addition, we kept the consensus sequences from the top 100 largest clusters. First, we quantified an unweighted percentage of identity between the GO terms for the consensus and the original sequences (UWGO100) through InterProScan searches. Then, we recalculated this parameter weighing by the number of original proteins per consensus sequence (WGO100), so as to be able to estimate the loss of sensitivity of GO terms recovery after clustering.

**Table 1.** Optimization of MMSEQS2 parameters for redundancy removal minimizing functional information

**-s**	**-c**	**—min-seq-id**	**cluster #**	**CR (%)**	**identity (%)**	**UWGO100 (%)**	**WGO100 (%)**
4.0	0.8	0.850	123 274	74.22	90.06	97.70	99.40
		0.900	133 699	72.04	92.89	97.72	99.80
		0.950	147 936	69.06	95.30	98.84	99.81
		0.975	159 438	66.66	96.44	98.77	99.82
		1.000	191 664	59.92	97.83	98.77	99.86
4.0	0.9	0.850	133 573	72.06	93.38	98.89	99.87
		0.900	142 106	70.28	95.50	100.00	99.35
		0.950	154 858	67.61	97.37	99.99	99.97
		0.975	165 711	65.34	98.26	98.81	99.92
		1.000	196 890	58.82	99.12	100.00	99.98
4.0	1.0	0.850	166 984	65.08	97.52	100.00	99.99
		0.900	169 958	64.46	99.00	100.00	100.00
		0.950	176 257	63.14	99.00	100.00	100.00
		0.975	183 176	61.69	99.48	100.00	100.00
		1.000	208 211	56.45	100.00	100.00	100.00
7.5	0.8	0.850	123 274	74.22	90.06	97.70	99.40
		0.900	133 698	72.04	92.89	97.72	99.80
		0.950	147 935	69.06	95.30	98.84	99.81
		0.975	159 435	66.66	96.44	98.77	99.82
		1.000	191 664	59.92	97.83	98.77	99.86

**-s** sensitivity MMSEQs2 parameter. Sets the average length of the lists of similar k-mers per query sequence position.

**-c** minimum coverage MMSEQS2 parameter. Only sequences are clustered that have a sequence length overlap greater than X% of the target sequence.

**—min-seq-id** MMSEQS2 parameter for minimum sequence identity. The number of identical aligned residues divided by the number of aligned columns including internal gap columns.

**CR** Database size reduction as percentage of clusters over the total number of input sequences.

**identity** Averaged percentage of identity between input and clustered sequences.

**UWGO100** Unweighted percentage of identity between the GO terms of the top 100 clusters with more input sequences.

**WGO100** Percentage of identity between the GO terms of the top 100 clusters with more input sequences, weighted by the number of sequences per cluster.

All the sequences from a cluster are aligned locally with mafft [44] to calculate the percentage of identity and the coverage. A consensus sequence for each cluster is then built with EMBOSS [45] and BLAST+, and DIAMOND databases are generated for the consensus sequence set. Two additional BLAST+ databases are constructed, one to store the *Arabidopsis* homologs of the consensus sequences obtained from TAIR10 using BLAST+, and another one containing known plant long non-coding RNA (lncRNA) sequences from CANTATA [46].

Once redundancy has been quantified and removed and there is a robust consensus set of protein sequences, the associated functional and metabolic information is collected from InterProScan and eggNOG-mapper using the parameters detailed in Supplemental Data S1. Besides the description, for each consensus protein sequence INTERPRO GO terms, PANTHER [47] GO terms, and MetaCYC metabolic pathway information is retrieved from InterProScan searches. In addition, ortholog sequence and species identification, orthologous groups of proteins and genes, eggNOG GO terms, E.C. numbers, KEGG KOs, pathways, modules, classes, reactions and T cell receptor signalling pathways, cazy, and pfam records are collected from eggNOG-mapper searches. Basic statistics of the consensus protein set are also calculated with NGShelper [15]: number of original proteins, number of consensus sequences, number of consensus sequences with functional annotation data collected from InterProScan and eggNOG-mapper searches, number of clusters with TAIR10 homologs and number of clusters without annotation. In addition, the completeness of the consensus protein set is assessed using BUSCO with a recently developed gene set for gymnosperms.

All the data, along with the BLAST+ and DIAMOND databases generated before, are then uploaded using scripts from NGShelper [15] into gymnotoa-db, which is a relational SQLite database (Supplemental Data S2) that is periodically updated by running the BASH script in an AWS r5.4xlarge instance and stored at the UPMdrive cloud repository (Universidad Politécnica de Madrid, 2014–2024). gymnotoa-db can be directly downloaded at https://blogs.upm.es/gymnotoa-db/gymnotoa-db/ or, preferably, through gymnotoa-app (https://github.com/GGFHF/gymnoTOA-app).

### gymnotoa-app software implementation and functioning

**Figure 2.**
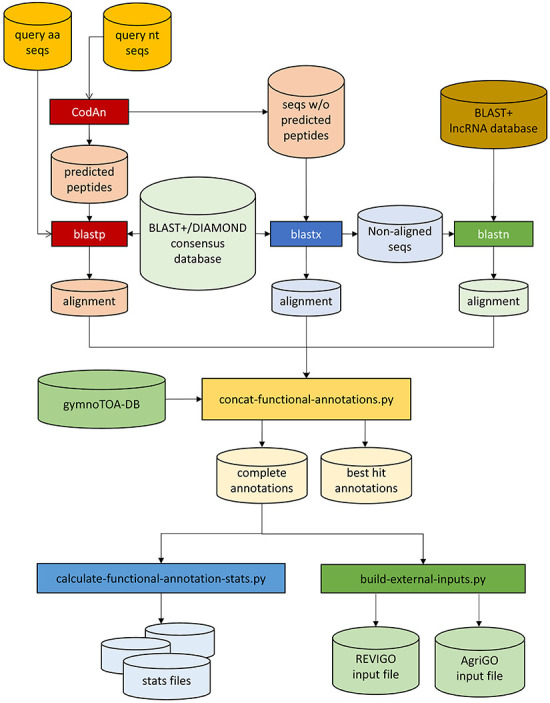
Workflow of GymnoTOA-APP.

gymnotoa-app was programmed in Python3 and can run either as a standalone application or through a user-friendly GUI in any computer with an OS supporting Python3: Linux/Unix, Windows, or macOS. gymnotoa-app employs the gymnotoa-db SQlite database and associated BLAST+ databases to extract functional information from multi-FASTA query nucleotide sequence files, such as those corresponding to ESTs/cDNA from RNAseq experiments or coding domains (CDs) from DNA-seq or whole genome sequencing experiments, or directly from protein FASTA files (Fig. 2). In the first case, nucleotide sequences are prompted to CodAn [48], so they are translated into amino acid sequences (query protein sequences). Homology searches are then performed against the BLAST+ or DIAMOND database for the consensus sequences from gymnotoa-db using *blastp* for the query protein sequences. In the case of nucleotide queries, for those nucleotide sequences not translated to amino acids by CodAn, homology searches are performed with *blastx*. The last homology search is performed using *blastn* with those sequences that did not show homology in the previous step against the lncRNA BLAST+ database from gymnotoa-db.

Once the homologous sequences have been identified, the associated functional and metabolic information for the best query-reference matches is then recovered and output in an easily readable .csv format, along with several graphics and output files ready to be used with REVIGO [49] or AGRIGO [50] to post-process the GO-terms obtained with gymnotoa-app. Furthermore, gymnotoa-app presents additional functionalities, such as functional enrichment analysis for sets of GO, KEGG, or MetaCYC terms Enriched functional terms are identified with the Fisher exact test [51] and either Benjamini–Hoghberg’s [52] or Benjamini–Yekutieli’s [53] FDR corrections with 0.05 significance level, using as background the functional terms collected in gymnotoa-db.

### Improvement of annotation using gymnotoa-db and gymnotoa-app

The improvement in sensitivity achieved by annotating gymnosperm transcript sequences with gymnotoa-app was estimated using a benchmark with 44 849 transcripts from a *de novo* assembled transcriptome of *Pinus canariensis* immature xylem [54] that was previously employed to test for TOA performance [39]. We compared gymnotoa-app with three similar applications employing different reference databases: (I) the original TOA using PLAZA Gymno01; (II) EnTAP using eggNOG; and (III) TRAPID using PLAZA Dicots 4.5.

In all cases, we estimated the following statistics, related to the sensitivity of the annotation: (I) total number of annotated sequences; (II) number of sequences with GO terms; (III) total number of GO terms found, and (IV) total number of non-redundant Biological Process (BP), Cellular Component (CC), and Molecular Function (MF) GO terms after redundant terms removal using semantic similarity (SimRel) with *rrvgo* Bioconductor package [55]. Intersection between sets of annotated transcripts and reduced GO-terms were visualized with Venn diagrams.

To test for improvement in accuracy we followed a gold-standard based procedure as in [56]. We first searched for all the gymnosperm proteins with a PDB record to ensure obtaining a robust set of proteins whose structure was determined experimentally by physical methods. This search resulted in 21 proteins, 15 of which showed a sequence record with “reviewed” status in the highly curated SwissProt database. The associated GO terms for the 15 sequences were retrieved from the links to GOA annotation records, which provide high-quality electronic and manual annotations of GO terms to UniProt Knowledgebase (UniProtKB) entries, and that were accessed through quickGO [57]. Then, the peptide sequences were translated to nucleotides using the Backtranseq utility from EMBOSS [45], and these sequences were used to simulate a degenerated small transcriptome with the debase-transcript-sequences.py script from NGShelper [15]. This script includes a probabilistic approach to fragment or shorten the original sequences and to introduce nucleotide substitutions and indels, so a more realistic *de novo* transcriptome can be simulated. We used a fragmentation probability of 30% (—fragprob = 0.3), allowing for three fragments per transcript (—maxfragnum = 3) of at least 50 nucleotides (--minfraglen=50) and a potential shortening of five nucleotides at most (--maxshortening=5). We also employed a single-nucleotide mutation probability of 50% (—mutprob = 0.5) and an indel mutation probability of 25% (—indelprob = 0.25) with a top of three mutations (—maxmutnum = 3) and a maximum of 10 nucleotides (—maxmutsize = 10). The fasta file for the small transcriptome used as a gold-standard had 22 transcripts, and is available with gymnotoa-app, along with a table of associated GO terms.

Functional annotation of the gold-standard transcriptome was performed using gymnotoa-app, TOA (PLAZA Gymno01), TRAPID (PLAZA Dicots 4.5), and EnTAP (EggNOG 5). The number of transcripts and proteins with assigned GO terms was determined for each tool. To reduce redundancy and remove obsolete terms, GO terms assigned by all tools were processed using REVIGO’s SimRel algorithm with medium (0.7) and small (0.5) similarity cutoffs, exploring the trade-off between retaining specific and general GO terms Global semantic similarity between each tool’s GO term set (BP, CC, MF) and the gold standard was then calculated using GoSemSim’s mgoSim function [58], using Wang’s similarity measure, the “rcmax” option, and the org.At.tair.db semantic data.

Finally, we also estimated runtimes and computational efficiency of all the annotation applications for the pine benchmark trascriptome. The runtimes for gymnotoa-app using BLAST+ or DIAMOND, TOA, and EnTAP were measured as the difference between end and start times of the pipeline with custom scripts. TRAPID runtimes are displayed in the application web page.

## Results

### Protein redundancy assessment

Original amino acid sequences retrieved from the NCBI server for Acrogymnospermae were 475 971. Using a combination of MMSEQS2 parameters to keep a 100% of identity between original sequences and clustered sequences (s = 4.0, c = 1.0 and -min-seq-id = 1.0), as well as keeping 100% of identical GO terms in the top 100 clusters with more input sequences than the original sequences forming the cluster, the original protein database was reduced by 56.45% after clustering (Table 1). In this case, 77.82% of the consensus sequences corresponded to a single original sequence, while 20.41% came from the clustering of 2–15 sequences and only 1.77% came from the clustering of more than 15 sequences. Other parameter combinations (s = 4.0, c = 1.0 and -min-seq-id = 0.9) reduced more substantially the number of redundant sequences (by 65.08%), but at the cost of reducing the average percentage of identity, the UWGO100 parameter and the percentage of non-annotated sequences. Indeed, a shift to more stringent clustering, from -min-seq-id = 1.0 to 0.9, resulted in a 2.88% increase in non-annotated consensus sequences, rising from 20% to 22.88%.

Functional annotation of consensus sequences derived from the selected parameters yielded annotation rates of 69.69% and 77.57% using InterProScan and eggNOG-mapper, respectively (Table 2). A total of 75.44% of the consensus sequences had an homolog in *Arabidopsis* (TAIR10). All gymnosperm conserved BUSCOs were found among the consensus sequences, evidencing good quality of the consensus dataset. Moreover, most of these BUSCOs were complete, with only two of them showing fragmentation.

**Table 2.** Basic statistics of the consensus protein database

	No. of consensus seqs	%
**Seqs annotated with InterProScan**	145 102	69.69^a^
**Seqs annotated with eggNOG-mapper**	161 510	77.57^a^
**Seqs with TAIR10 orthologs**	157 078	75.44^a^
**Seqs w/o annotation**	41 645	20.00^a^
**Complete BUSCOs**	1601	99.88^b^
**Fragmented BUSCOs**	2	0.12^b^
**Missing BUSCOs**	0	0^b^

Absolute number of sequences and percentage over all sequences is indicated.

a Percentage over 208 211 consensus sequences.

b Percentage over 6103 gymnosperm BUSCOs.

### Improvement of transcriptome annotation with gymnotoa-db and gymnotoa-app

**Figure 3.**
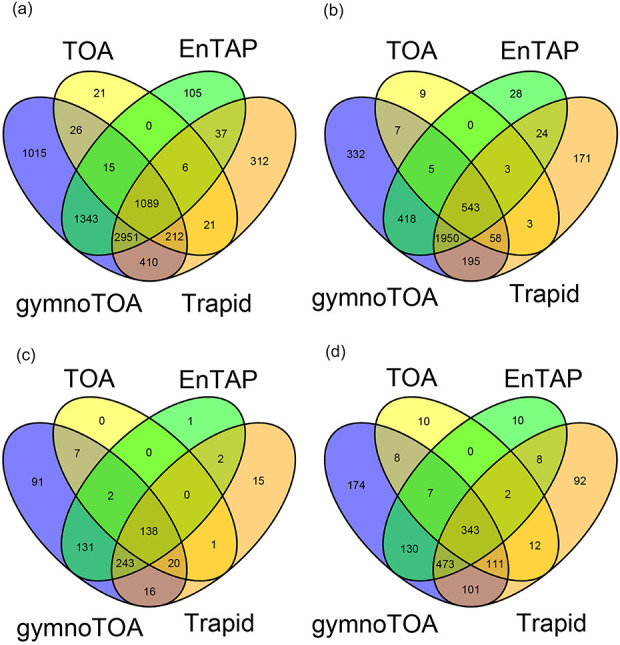
Venn diagrams for numbers of annotated sequences and recovered GO terms for a xylogenesis transcriptome (44 849 transcripts) of *Pinus canariensis* with gymnotoa-app using BLAST+ (gymnotoa-db), TOA (PLAZA Gymno01), EnTAP (eggNOG), and TRAPID (PLAZA Dicots 4.5). For visualization purposes, we do not show the results from gymnotoa-db using DIAMOND. Unique GO term numbers correspond to the best hit sequence. GO term numbers by BP, CC, and MF are obtained after computing the similarity matrix between GO terms and reducing redundant GO terms based upon the genome wide annotation database for *Arabidopsis*, org.At.tair.db. (A) all GO terms; (B) GO terms for BP; (C) GO terms for CC; (D) GO terms for MF.

The sensitivity of gymnotoa-app using the *Pinus canariensis* benchmark transcriptome differed slightly depending on the homology search algorithm employed. Functional information for 32 433 transcripts (72.32%) and GO terms for 27 770 of them was obtained using BLAST + . Using DIAMOND, less transcripts could be annotated (30 714 transcripts, 68.5%), and consequently, less transcripts presented GO terms (26 095). Considering only the results obtained using BLAST+, gymnotoa-app produced better results than eggNOG (EnTAP) and PLAZA Dicots 4.5 (TRAPID) both in terms of annotated sequences and of recovery of unique and non-redundant GO terms (Table 3). If compared to PLAZA Gymno01 (TOA), the results showed a similar number of sequences with functional information of at least one of the gene ontologies provided by TOA and gymnoTOA, which are more complete than in the other applications. Nevertheless, gymnotoa-app recovered 7061 unique GO terms, with 1015 GO terms that were not identified with the other applications (Fig. 3a), outperforming all the other applications at this respect. A consistent pattern of GO term recovery was found considering GO terms from BP (Fig. 3b), CC (Fig. 3c), and MF (Fig. 3d) alone. In all cases, gymnotoa-app retrieved significantly more exclusive GO terms than the other applications (344 for BP, 94 for CC, and 167 for MF). TRAPID using PLAZA Dicots 4.5 was the second application that recovered more exclusive GO terms (163 for BP, 15 for CC, and 84 for MF). Most GO terms retrieved with TOA (PLAZA Gymno01) and EnTAP (eggNOG) were also recovered by gymnotoa-app for all GO term categories (Fig. 3).

**Table 3.** Comparison of annotation sensitivity and runtimes of a xylogenesis transcriptome (44 849 transcripts) of *Pinus canariensis* with gymnotoa-app (gymnotoa-db using BLAST+ and DIAMOND), TOA (PLAZA Gymno01), EnTAP (eggNOG), and TRAPID (PLAZA Dicots 4.5)

Statistics	gymnoTOA (BLAST+)	gymnoTOA (DIAMOND)	TOA	EnTAP (runP)	TRAPID
**No. of annotated seqs**	32 433	30 714	32 589	19 957	32 155
**No. of seqs w/ GO terms**	27 770	26 095	29 523	14 750	24 518
**No. of unique GO terms**	7061	7011	1390	5546	5038
**BP—rrvgo**	3508	3479	628	2971	2947
**CC—rrvgo**	648	645	168	517	435
**MF—rrvgo**	1347	1339	493	973	1142
**Testing** **platform**	VMware (Ubuntu 24.04-4CPUs-16GB) on Windows11 (i9-11950 H)			AWS r5.4xlarge (16CPUs-128GB)	Web server
**Runtime**	02:46:33	00:03:05	06:38:50	01:15:29	01:37:47

gymnoTOA and TOA were run on a virtual machine VMware (Ubuntu 24.04–4CPUs-16GB) on W10 (i9-11950 H); EnTAP was run on an AWS r5.4xlarge instance with 16 CPUs-128GB); TRAPID was run on the web server of the VIB-UGENT Center for Plant Systems cluster facilities. In all cases, the *e*-value threshold in homology searches was 10^–5^. Unique GO term numbers correspond to the best hit sequence. GO term numbers by BP, CC, and MF are obtained after computing the similarity matrix between GO terms and reducing redundant GO terms based upon the genome wide annotation database for *Arabidopsis*, org.At.tair.db

In addition to its greater sensitivity, gymnotoa-app proved to be more accurate than the other functional annotation applications, getting the same results for the gold-standard regardless of using BLAST+ or DIAMOND (Table 4). While TOA, TRAPID and EnTAP were unable to find homology for 5, 6, and 11 transcripts, respectively, gymnotoa-app was able to find associated functional annotation for all but one transcript. To enable comparison, semantic similarity to the gold standard was calculated only for transcripts homologous across all applications. After removing obsolete and redundant GO terms using REVIGO with relaxed (cutoff = 0.7) and stringent (cutoff = 0.5) thresholds, gymnotoa-app achieved the highest average similarity (BP, CC, MF) with the relaxed threshold (0.800) and the second-highest (after TRAPID) with the stringent threshold (0.773).

**Table 4.** Functional annotation accuracy comparison of the gold-standard transcriptome with gymnotoa-app (gymnotoa-db using BLAST+), TOA (PLAZA Gymno01), EnTAP (eggNOG), and TRAPID (PLAZA Dicots 4.5)

	gold-standard	gymnoTOA (BLAST+)^a^	TOA	TRAPID	EnTAP (runP)
**No. of Transcripts/prots w/o GO**	0/0	1/1	5/4	6/4	11/6
**No. of unique GO terms**	62	276	31	323	247
**No. of unique GO terms prots annotated all pipelines**	35	182	21	248	199
**No. of GO-REVIGO**	30 (25)	120 (85)	17 (17)	150 (110)	131 (100)
**No. of BP-revigo**	11 (8)	81 (52)	4 (4)	91 (62)	77 (52)
**No. of CC-revigo**	3 (3)	18 (15)	2 (2)	28 (22)	14 (12)
**No. of MF-revigo**	16 (14)	21 (18)	11 (11)	31 (26)	40 (36)
**Semantic similarity-BP**	1.000 (1.000)	0.617 (0.583)	0.579 (0.579)	0.585 (0.604)	0.557 (0.552)
**Semantic similarity-CC**	1.000 (1.000)	1.000 (1.000)	0.686 (0.686)	0.965 (0.965)	0.818 (0.818)
**Semantic similarity-MF**	1.000 (1.000)	0.774 (0.736)	0.787 (0.766)	0.842 (0.810)	0.700 (0.687)
**Semantic similarity-average**	1.000 (1.000)	0.800 (0.773)	0.687 (0.677)	0.797 (0.793)	0.692 (0.686)

The table presents: (i) the number of transcripts/proteins lacking GO term annotation; (ii) the number of unique GO terms assigned by each tool; (iii) the number of GO terms only for the annotated proteins with all tools; and (iv) the total number of GO terms (BP, CC, and MF) after filtering with REVIGO (cutoff = 0.7 and 0.5, the latter among brackets). Semantic similarity scores (overall and for each GO namespace) are also provided.

a The results for gymnotoa-app using DIAMOND and BLAST+ are identical.

Regarding computation runtimes, the performance comparison between applications is not straightforward, because they run on different hardware systems (Table 3). gymnotoa-app run on a standard VM of 4 cores, 16 GB RAM in only a few minutes using DIAMOND with default parameters and in <3 h using BLAST + . These results significantly outperform those from running TOA using the same hardware system. For EnTAP, however, runs could not be completed using the same hardware, and a more powerful AWS r5.4xlarge (16CPUs-128GB) was used instead in just over an hour. TRAPID, on the other hand, presents similar times to EnTAP, since it runs in remote, using the web server of the VIB-UGENT Center for Plant Systems cluster facilities.

## Discussion

gymnotoa-db is a public resource that stores an up-to-date set of unique non-redundant protein sequences along with complete and robust functional information for those sequences. Through an automatic procedure run in the AWS cloud, which is implemented to build the database, researchers focused on transcriptomics or genomics are released of having to program complicated pipelines to search and optimize reference sequences and their associated functional information. Moreover, gymnotoa-db can be accessed with gymnotoa-app, a companion application that can be operated in Linux, Windows or macOS and that has proven to increase sensitivity and accuracy of transcript functional annotation in gymnosperms as compared to other similar functional annotation software tools that exploit the information from other publicly available databases.

Protein databases grow at an astronomical rate, together with the redundancy of their entries [59]. A small part of redundant sequences stored at NCBI may be due to errors in curation of erroneous entries [60], but the majority of redundancy is produced by contamination or sequence duplication. Contamination occurs mostly by incorrectly labeled reference sequences [61]. Sequence duplication may arise in a variety of ways and its impact on downstream analysis will depend on the context [37]. gymnotoa-db improves functional annotation by mitigating the impact of duplicate protein records arising from: (I) orthologous genes sharing identical or nearly identical sequences across species; (ii) paralogous genes encoding identical proteins but represented by separate entries; and (iii) distinct entries for analogous gene family members or isoforms.

We have employed a method based on maximizing identity similarity and functional annotation data consistency to remove duplicates by clustering using MMSEQS2 with a combination of optimal parameters to keep 100% of identity between original sequences and clustered sequences, as well as keeping 100% of identical GO terms in the top 100 clusters with more input sequences than the original sequences forming the cluster. Optimized sequence clustering using MMSEQS2 generated a representative set of consensus sequences, often grouping homologous sequences from different gymnosperm species. This parameter combination balances database size and functional annotation completeness. While MMSEQS2 significantly reduced the number of input sequences, over 75% of consensus sequences represent a single input sequence. The remaining consensus sequences derived from clusters containing more than two redundant sequences. This reduction minimizes hardware requirements without compromising functional information. More stringent clustering parameters, while potentially further reducing database size, could negatively impact functional annotation by generating suboptimal, potentially chimeric, consensus sequences.

For this curated set of gymnosperm protein sequences, gymnotoa-db offers more complete functional information compared to the databases underlying other functional annotation applications, as it incorporates functional information for several ontology systems, such as GO, KEGG, E.C., MetaCYC metabolic pathways, etc., collected not only from InterProScan, but also from eggNOG-mapper. While InterProScan provides functional analysis of proteins by classifying them into families and predicting domains and important sites [32], eggNOG-mapper is based on fast orthology assignments using pre-computed clusters and phylogenies [33]. This dual functional annotation search of the set of gymnosperm non-redundant sequences allows to store more complete functional meta-information in gymnotoa-db than in other similar databases. In addition, gymnotoa-db provides the sequence homologs in Arabidopsis and contains also plant lncRNA registries with regulatory function that will not code for proteins from CANTATA [46] that can be accessed using the companion gymnotoa-app utility. Notwithstanding, while these results of accuracy are positive, it is worth mentioning the presence of *∼*20% of non-annotated sequences in gymnotoa-db. Currently, the existing genomic resources for gymnosperms are by far much more scarce if compared to other plant model species [3], and this still hampers a robust functional annotation in gymnosperms However, the percentage of non-annotated proteins will hopefully decrease as the research community adds genomic and transcriptomic resources from gymnosperm experiments to the NCBI Protein database.

Despite these limitations, the comparison of functional annotation results revealed higher sensitivity and accuracy of gymnotoa-app with respect to other functional annotation applications that explore other databases. All of them translate transcripts into amino acid sequences to then performed homology searches against cross-referenced databases that include a set of reference amino acid sequences and associated functional information. On the one hand, gymnotoa-db improved sensitivity by annotating more transcripts and recovering more functional information from several sources and ontology systems than other applications, especially when operated with BLAST + . Indeed, while it took only a few minutes to annotate a standard pine transcriptome of nearly 45 000 transcripts using gymnotoa-app with DIAMOND default parameters instead of BLAST+, almost 2000 less transcripts were annotated in the former case. These results agree with what was previously reported for TOA [39]. Actually, while validating TOA, BLAST+ based pipelines were able to annotate *c*. 1.7% more sequences than those using DIAMOND. Moreover, focusing on the most widely used functional ontology, i.e. Gene Ontology, gymnotoa-app showed a more complete recovery of unique and non-redundant GO terms with respect to the other reference datasets. However, the GUI of gymnotoa-app provides the option to modify DIAMOND parameters, enabling users to prioritize sensitivity at the expense of computational performance.

On the other hand, in addition to the good results obtained for the benchmark pine transcriptome, functional information was also obtained for more gold-standard transcripts with gymnotoa-app than with other applications. This result may be partially explained by the specificity of the gymnosperms consensus sequences in gymnotoa-db, but also by a more reliable translation of transcripts to amino acids in gymnotoa-app with CodAn than in the other applications that employ other methods, such as TransDecoder [62]. Irrespective of using one or another application, the recovered functional information was also more reliable using gymnotoa-app than other applications, as shown by the improvement in accuracy reported for the gold standard. In functional annotation of gymnosperms, a standard accuracy assessment of false positives and negatives is not easy to implement because of lack of uniformity in the descriptions of the records in the different databases [63] and of the redundant hierarchical nature of GO records [64]. Our approach to assess accuracy was based on estimating semantic similarity of GO terms sets retrieved for a gold-standard transcriptome with different functional annotation applications [56]. However, the lack of experimentally annotated proteins in gymnosperms [3] and the consequential small set of transcripts that composed the gold-standard may bias accuracy estimation, and therefore, the improvement in accuracy should be considered with caution.

Even with the aforementioned limitations, our results point to a better recovery of unique features of gymnosperm amino acid sequences in gymnotoa-db than in the other databases. gymnotoa-db recovered most of the functional information from eggNOG (EnTAP) and outperformed the outdated PLAZA gymno01 database (TOA). It also produced more complete results than PLAZA Dicots 4.5 (TRAPID), which is the database that included more exclusive GO terms after gymnotoa-db.

In summary, gymnotoa-db and gymnotoa-app are useful resources to easily obtain up-to-date and reliable functional information from several widely used ontology systems in transcriptomics and genomics experiments focused on gymnosperms. This contributes to partly alleviate the problem of the lack of genomic resources in gymnosperms and facilitates their bioinformatics analysis. The automatic database construction and fast and user-friendly associated application is intended to release scientists working with gymnosperms and without strong bioinformatic background of performing sequence fetching, redundancy removal, and functional annotation data search with sometimes complicate algorithms.

## Supplementary Material

baaf019_Supp

## Data Availability

gymnotoa-db is available for download at https://blogs.upm.es/gymnotoa-db/gymnotoa-db/; gymnotoa-app is available for download at https://blogs.upm.es/gymnotoa-db/gymnotoa-db/ and at a GitHub repository ((https://github.com/GGFHF/gymnoTOA-app) along with a manual describing installation and functioning recipes, including the script to generate gymnotoa-db.
