# Correction to: GymnoTOA-db: a database and application to optimize functional annotation in gymnosperms

**DOI:** 10.1093/database/baaf041

**Published:** 2025-06-26

This is a correction to: Fernando Mora-Márquez, Mikel Hurtado, Unai López de Heredia, GymnoTOA-db: a database and application to optimize functional annotation in gymnosperms, *Database*, Volume 2025, 2025, baaf019, https://doi.org/10.1093/database/baaf019.

The link to the gymnoTOA database has been updated throughout this paper to https://blogs.upm.es/gymnotoa-db/gymnotoa-db/.

