# Supplementary material for: gymnotoa-db: a database and application to optimize functional annotation in gymnosperms
Source: Database (Oxford). 2025 Mar 6;2025:baaf019. doi: 10.1093/database/baaf019 (PMC11886576; doi:10.1093/database/baaf019)
Supplement: baaf019_Supp [file baaf019_Supp.zip › suppl_data/SupplData-S1-InterProScan-eggNOGmapper-parameters-R2.docx]

**Supplemental Data S1:** Parameters used to run InterProScan (Table 1) and eggNOG-mapper (Table 2) on consensus sequences from gymnoTOA-db.

**Table 1:** InterProScan parameters.

| **Parameter** | **Value** |
| --- | --- |
| --cpu | 16 |
| --input | path of consensus sequences file |
| --seqtype | p |
| --applications | AntiFam,CDD,Coils,Gene3D,Hamap,MobiDBLite,  NCBIfam,PANTHER,Pfam,PIRSF,PIRSR,PRINTS,  ProSitePatterns,ProSiteProfiles,SFLD,SMART,  SUPERFAMILY,TIGRFAM |
| --iprlookup |  |
| --goterms |  |
| --pathways |  |
| --formats | TSV |
| --output-dir | path of temporal directory |

**Table 2:** eggNOG-mapper parameters.

| **Parameter** | **Value** |
| --- | --- |
| --cpu | 16 |
| -i | path of consensus sequences file |
| --itype | proteins |
| -m | diamond |
| --dmnd_algo | auto |
| --sensmode | sensitive |
| --dmnd_iterate | yes |
| --evalue | 0.00001 |
| --output_dir | path of temporal directory |
| --output | Acrogymnospermae-consensus |
