# Supplementary material for: gymnotoa-db: a database and application to optimize functional annotation in gymnosperms
Source: Database (Oxford). 2025 Mar 6;2025:baaf019. doi: 10.1093/database/baaf019 (PMC11886576; doi:10.1093/database/baaf019)
Supplement: baaf019_Supp [file baaf019_Supp.zip › suppl_data/SupplData-S2-SQLite-DATABASE-Description.pdf]

Supplemental Data S2: Structure of gymnoTOA-DB. The database consists of five tables for which description of text fields and relational indices are shown.

Table 1: mmseq2_relationships

Column	Type	Index	Comment
cluster_id	TEXT	1	cluster identification
seq_id	TEXT	2	NCBI protein sequence identification
description	TEXT		description from the NCBI protein sequence
species	TEXT		species from the NCBI protein sequence

Table 2: interproscan_annotations

Column	Type	Index	Comment
cluster_id	TEXT	1	cluster identification
interpro_goterms	TEXT		concatenated list of GO terms from InterPro
panther_goterms	TEXT		concatenated list of GO terms from Panther
x_goterms	TEXT		concatenated list of GO terms from other sources
metacyc_pathways	TEXT		concatenated list of pathway identifications from MetaCyc
reactome_pathways	TEXT		concatenated list of pathway identifications from Reactome
x_pathways	TEXT		concatenated list of pathway identifications from other sources

Table 3: emapper_annotations

Column	Type	Index	Comment
cluster_id	TEXT	1	cluster identification
ortholog_seq_id	TEXT		ortholog sequence identification from eggNOG
ortholog_species	TEXT		species from eggNOG
eggnog_ogs	TEXT		OGs (Orthologous Groups) of proteins from eggNOG
cog_category	TEXT		COG (Cluster of Orthologous Genes) from eggNOG
description	TEXT		description from eggNOG
goterms	TEXT		concatenated list of GO terms from eggNOG
ec	TEXT		concatenated list of EC (Enzyme Commission) numbers
kegg_kos	TEXT		concatenated list of KO from KEGG
kegg_pathways	TEXT		concatenated list of pathway identifications from KEGG
kegg_modules	TEXT		concatenated list of module identifications from KEGG
kegg_reactions	TEXT		concatenated list of chemical reactions identifications from KEGG
kegg_rclasses	TEXT		concatenated list of reactions classification identifications from KEGG
brite	TEXT		functional hierarchy of OGs assigned to the sequence
kegg_tc	TEXT		T cell receptor (TCR) signaling pathway
cazy	TEXT		concatenated list of Carbohydrate-Active Enzymes (CAZymes)
pfams	TEXT		concatenated list of protein families from Pfam

Table 4: tair10_orthologs

Column	Type	Index	Comment
cluster_id	TEXT	1	cluster identification
ortholog_seq_id	TEXT		ortholog sequence identification of *A. thaliana*

Table 5: go_ontology

Column	Type	Index	Comment
go_id	TEXT	1	GO term identification
go_name	TEXT		GO term description
namespace	TEXT		molecular function, biological process or cellular component
